# Remote monitoring in implantable cardiac devices: A care solution or alert pollution?

**DOI:** 10.1007/s12471-025-01957-0

**Published:** 2025-05-05

**Authors:** Cheyenne S. L. Chiu, Mathias Meine

**Affiliations:** Department of Cardiology, Division Heart & Lungs, University Medical Centre Utrecht, Utrecht, The Netherlands

The management of patients with cardiac implantable electronic devices (CIEDs) has undergone a fundamental shift in recent years. Previously considered as merely an adjunct to in-clinic follow-up, remote monitoring (RM) is now an integral part of device management. The COVID-19 pandemic served as a catalyst, highlighting its ability to effectively replace routine clinic visits while maintaining safety [1]. More than just a substitute for visits, RM facilitates early detection of arrhythmias and device malfunctions, reduces healthcare costs, and is associated with high patient satisfaction [2, 3]. Despite these established benefits, RM adoption remains low among pacemaker patients, probably due to limited research in this population, as most studies have focused on ICD patients.

In this issue of the *Netherlands Heart Journal*, Adriaansen et al. present the HERO study, a retrospective, single-center analysis of RM in pacemaker patients aged ≥ 18 years with elevated stroke risk (CHA_2_DS_2_-VASc score ≥ 2), no history of atrial fibrillation or atrial flutter, and a dual- or triple-chamber pacemaker [4]. In total, 203 participants were included, with 60 patients receiving RM alongside routine clinic visits (RM+), and 143 monitored exclusively via clinic visits (RM−). Over a median follow-up of 5.0 years, no significant differences were observed in the time to the first atrial or ventricular arrhythmic event, device-related adverse events, or anticoagulation adjustments.

As noted by the authors, these findings contrast with the RAPID and SETAM studies, which showed that RM enables earlier arrhythmia detection in pacemaker patients. However, the HERO study has notable methodological limitations. Its retrospective design introduced significant baseline differences between the study groups, making result interpretation more challenging. Selection bias was evident, as patients on anticoagulation were rarely offered RM due to the limited clinical impact of early atrial arrhythmia detection. Furthermore, heart failure and cardiac resynchronization therapy pacemakers were more frequent in the RM− group. All RM+ patients were implanted with Biotronik pacemakers, whereas the RM− group included both Biotronik and Boston Scientific devices. Moreover, the median time from implantation to RM acceptance was 76 days, with RM offered at either the two-week follow-up or annual visit. Kaplan-Meier analysis indicated higher event detection in RM+ patients within the first 1.5 years. It is conceivable that initiating RM at discharge could further improve early arrhythmia detection.

Although the HERO study did not demonstrate a significant reduction in time to arrhythmia detection, it confirmed the feasibility of RM with high patient acceptance and minimal technical malfunctions. It also highlighted a substantial arrhythmia burden, with 53.7% of participants experiencing at least one event. However, the clinical relevance of this burden may be overstated, as 79.8% of the first events were atrial tachyarrhythmias, of which 29.4% lasted less than six minutes. These brief episodes are not considered to increase stroke risk and do not require anticoagulation. The clinical implications of atrial high-rate episodes between six minutes and 24 h remain uncertain. HERO patients with such episodes were included in either the ARTESIA or NOAH-AFNET 6 trial. The ARTESIA study found that apixaban reduced the risk of stroke and embolism but increased major bleeding [5], whereas NOAH-AFNET 6 showed no cardiovascular benefit and a higher risk of major bleeding and mortality with edoxaban [6].

Meanwhile, the study raised concerns about clinical workload, as the RM+ group required more telephone consultations without a reduction in clinic visits. Despite this increased healthcare burden, the authors suggest that RM, if implemented efficiently, could ultimately reduce this burden. The COMPAS randomised controlled trial has already established that RM is a safe alternative to routine clinic visits and significantly lowers the number of visits in pacemaker patients [7]. However, with RM generating vast amounts of data—most of it not clinically relevant—it risks overloading clinicians. Therefore, the real question is not whether RM can replace clinic visits, but how to streamline data management without overwhelming clinical workflows.

Artificial intelligence (AI) could be the key to solving this challenge by filtering out irrelevant alerts, prioritizing urgent cases, and automating routine management tasks. Additionally, integrating AI into a smartphone-based application could enable automated communication. This would not only provide real-time feedback on monitoring results but also let patients input relevant symptoms, supporting bidirectional communication. This may allow healthcare providers to focus on patient care rather than handling excessive notifications.

As interest in real-time monitoring grows, wearables are increasingly embraced for health tracking. Yet, CIEDs already contain advanced built-in sensors capturing physiological data that remains underutilized. RM can be more than just a safety net for arrhythmic events and device issues. AI-driven predictive modeling using continuous CIED sensor data could enable early detection of subtle physiological and technical changes, facilitating timely intervention and reducing adverse events. In an era of rapid technological advancement, RM should no longer be seen merely as an adjunct or substitute for routine follow-up visits—it represents the future of personalised, predictive, and proactive cardiovascular care.

## References

[1] Chiu CSL, Timmermans I, Versteeg H, et al. Effect of remote monitoring on clinical outcomes in European heart failure patients with an implantable cardioverter-defibrillator: secondary results of the REMOTE-CIED randomized trial. Europace. 2022;24:256–67. 34410384 10.1093/europace/euab221 PMC8499745

[2] de Graaf G, Timmermans I, Meine M, et al. Economic evaluation of remote monitoring of patients with an implantable cardiac defibrillator (REMOTE-CIED study). J Telemed Telecare. 2024;30:1173–85. 36245363 10.1177/1357633X221129176

[3] Timmermans I, Meine M, Szendey I, et al. Remote monitoring of implantable cardioverter defibrillators: Patient experiences and preferences for follow-up. Pacing Clin Electrophysiol. 2019;42:120–9. 30536931 10.1111/pace.13574 PMC6849564

[4] Adriaansen F, Seelig J, de Vries T, et al. HEart Rhythm management Optimization of pacemaker recipients using remote monitoring: the HERO registry. Neth Heart J. 2025; 10.1007/s12471-025-01953-4. 10.1007/s12471-025-01953-4 PMC12098215 40343620

[5] Healey JS, Lopes RD, Granger CB, et al. Apixaban for Stroke Prevention in Subclinical Atrial Fibrillation. N Engl J Med. 2024;390:107–17. 37952132 10.1056/NEJMoa2310234

[6] Kirchhof P, Toennis T, Goette A, et al. Anticoagulation with Edoxaban in Patients with Atrial High-Rate Episodes. N Engl J Med. 2023;389:1167–79. 37622677 10.1056/NEJMoa2303062

[7] Mabo P, Victor F, Bazin P, et al. A randomized trial of long-term remote monitoring of pacemaker recipients (The COMPAS trial). Eur Heart J. 2012;33:1105–11. 22127418 10.1093/eurheartj/ehr419 PMC3341630

